# High-risk biochemical recurrence in prostate cancer: identification and early intervention strategies

**DOI:** 10.3389/fruro.2026.1719136

**Published:** 2026-01-29

**Authors:** Ahmed Hassan Abdelaziz, Mai Mohamed Ali Ezz El Din, Emad Hamada, Mohamed Abdullah, Hassan Sayed Shaker, M. Sherif Mourad, Tarek Osman, Amr Mohamed Nowier, Dina Salah Gad

**Affiliations:** 1 Ain Shams University, Cairo, Egypt; 2 Cairo University, Cairo, Egypt; 3 Astellas, Cairo, Egypt

**Keywords:** androgen receptor signaling inhibitors, biochemical recurrence, early detection, Egypt, multidisciplinary approach, prostate cancer

## Abstract

**Purpose:**

Biochemical recurrence (BCR) following primary therapy for prostate cancer (PCa) is associated with disease progression; thus, identifying patients at high risk and implementing management strategies remains critical. This expert opinion outlines a set of recommendations for identifying high-risk BCR patients, provides insights into the impact of a multidisciplinary team (MDT) approach on disease management, explores associated costs and resource utilization, and examines the role of androgen receptor signaling pathway inhibitors (ARPIs) in optimizing outcomes.

**Methods:**

The latest evidence and clinical guidelines on risk stratification, diagnostic tools, and collaborative management strategies were evaluated. Additionally, expert opinions were collected from nine oncology and urology experts, and their insights were integrated to form a comprehensive approach tailored for clinical application.

**Results:**

The panelists reached agreement on several proposed questions, including patients’ early detection, risk stratification, early management, and the role of ARPIs and androgen deprivation therapies (ADT). The recommendations emphasize the need for standardized identification of high-risk BCR patients, treatment protocols, and early intervention strategies. Additionally, the multidisciplinary approach facilitates personalized treatment planning, leveraging various specialties’ expertise, and addresses the complexity of resource utilization and cost management. However, a lack of agreement on other topics was observed, such as optimal timing of intervention and resource allocation strategies.

**Conclusion:**

This narrative, evidence-supported expert-opinion review highlights the importance of standardized protocols, multidisciplinary strategies, and the integration of advanced diagnostics and androgen receptor pathway inhibitors to improve patient outcomes. Further research is warranted to refine predictive models, optimize resource allocation, and enhance therapeutic efficacy.

## Highlights

• The lack of a universally accepted definition for BCR complicates treatment initiation decisions.
• Techniques like Ga. PSMA.PET/CT scans enhance early and accurate detection of BCR.
• Early intervention improves survival outcomes, though further research is needed.
• Treatment decisions should balance long-term benefits with potential toxicity, tailored to individual patient profiles.

## 1 Introduction

Prostate cancer (PCa) remains a significant global health challenge, with around 190,000 new cases diagnosed annually and 80,000 mortalities worldwide (1). In Egypt, the five-year prevalence of PCa in 2022 was 11,541 cases, making it the 8^th^ most common cancer (2). Despite treatment, patients develop prostate-specific antigen (PSA) recurrence, known as biochemical recurrence (BCR), with a recurrence risk of 20-40% (3). Therefore, early identification of those patients is crucial, particularly those with BCR following initial treatment. The lack of agreement on the BCR definition complicates early intervention and management strategies (4–6). Despite advancements in therapeutic options, including androgen deprivation therapy (ADT)-sparing regimens, and androgen receptor signaling pathway, the treatment strategies are not well established (7). The choice of initial definitive treatment, whether radical prostatectomy (RP) or radiation therapy (RT), influences both the definition and timing of BCR. For example, BCR after RP is often defined by a PSA threshold > 0.2 ng/mL, while after RT, a rise of > 2 ng/mL above the PSA nadir is considered BCR. However, different definitions of disease progression influence treatment decisions (8). Moreover, the choice to treat BCR requires careful consideration of the burden or toxicity associated with overtreatment.

The Egyptian expert panel addressed the pressing clinical challenge of high-risk biochemical recurrence (BCR) in prostate cancer, with a focus on clear translational relevance for real-world urology and oncology practice. This panel uniquely integrates multidisciplinary perspectives by including both urologists and oncologists, fostering comprehensive, multidisciplinary team (MDT)-oriented recommendations tailored to regional clinical needs and practice realities.

Accordingly, these recommendations aim to identify high-risk BCR patients, explore patient preferences for ADT-sparing regimens, evaluate the role of androgen receptor signaling pathway inhibitors (ARPIs) as monotherapy in non-metastatic castration-sensitive prostate cancer (nmCSPC), assess the impact of multidisciplinary approaches on early detection, and identify associated costs and healthcare resource utilization.

## 2 Methodology

### 2.1 Advisory board composition

The panel comprised nine Egyptian experts: four clinical oncologists and five urologists, with expertise in managing patients with PCa. Experts’ selection criteria include the relevance of experience, experience in PCa research, affiliation with reputable institutions, membership in multidisciplinary collaborations, and contribution to guidelines development.

### 2.2 Scope and process

A literature review was conducted on databases such as PubMed, EMBASE, and The Cochrane Library to identify gaps and unmet needs from 2014 to 2024 (**Table 1**). Afterward, a 29-question questionnaire was developed to identify high-risk patients, explore patient preferences for ADT-sparing regimens, evaluate ARPIs as monotherapy, assess the impact of multidisciplinary approaches, and identify associated costs and resource utilization.

**Table 1:** Literature selection criteria.

Inclusion criteria	Exclusion criteria
Studies published between 2014 and 2024 to align with the panel’s review timeframe.	Publications before 2014 to maintain recency.
Articles addressing biochemical recurrence (BCR) in prostate cancer (PCa), including definitions, risk factors, high-risk identification, and management strategies post-radical prostatectomy (RP) or radiation therapy (RT).	Studies unrelated to BCR in PCa, such as primary prevention, screening without recurrence focus, or non-prostate cancers.
Research on ADT-sparing regimens, androgen receptor pathway inhibitors (ARPIs) in non-metastatic castration-sensitive PCa (nmCSPC), multidisciplinary team (MDT) approaches, imaging (e.g., PSMA-PET/CT), PSA doubling time (PSA-DT), and resource utilization.	Non-peer-reviewed sources like editorials, letters, abstracts, case reports, or animal/preclinical studies lacking human clinical data.
Full-text articles available in English from PubMed, EMBASE, or Cochrane Library, focusing on clinical trials, guidelines, expert consensus, or observational studies relevant to Egypt or Middle East/North Africa contexts.	Articles without full-text access, non-English language, or irrelevant to high-risk BCR, ADT-sparing therapies, or regional healthcare challenges in Egypt.

Eight expert responses to all questions were collected anonymously from September 8th to 30^th^, 2024, using the LimeSurvey platform. They were primarily urologists, but three oncologists’ insights were also included. The data collected was analyzed to identify key trends, correlations, and outliers. In October 2024, a session was held in Cairo, Egypt, to reach expert opinion recommendations. During the meeting, experts reviewed the questionnaire results and shared insights about each section. All disagreements were handled during the meeting, and the panel agreed on the final voting results. One panel member attended the meeting but did not participate in the voting process.

### 2.3 Voting and discussion

This expert opinion includes both quantitative and qualitative questions to assess agreement and incorporate insights from clinical practice. The quantitative questions were analyzed using a five-point Likert scale. For responses analysis, ‘agree’ and ‘strongly agree’ responses were considered as agreement, while ‘disagree’ and ‘strongly disagree’ were considered as disagreement. Levels of agreement were categorized as less than 37%, 37%-67%, 67%-87%, and more than 87%, indicating no agreement, slight agreement, agreement, and strong agreement, respectively.

## 3 Results and discussion

### 3.1 Applicability of international guidelines

#### 3.1.1 Reliance on international guidelines and their limitations

Panelists relied on international guidelines for diagnosing and managing PCa; however, critical gaps were noticed, including a lack of agreement on defining high-risk PCa and the absence of standardized treatment protocols for high-risk BCR patients. Furthermore, the panelists noted that Egypt’s healthcare infrastructure and resource limitations significantly hinder guidelines implementation. This underscores the need to develop a local expert opinion to optimize PCa management in Egypt.

#### 3.1.2 Current initiatives for local guidelines and barriers

Some national institutions have developed local Egyptian PCa screening and diagnosis guidelines, such as the Egyptian Urological Association (EUA) Guidelines on Prostate Cancer, produced as an initiative of the Supreme Council of University Hospitals to collaborate with the Egyptian Society of Urological Research (ESURS) (9). However, only 25% of panelists implemented these guidelines due to the lack of awareness and clinical governance. Moreover, the absence of national registries and proper documentation impedes tailoring national recommendations to Egypt’s needs. The panel suggests establishing a national cancer registry to encourage full documentation essential for addressing gaps and adapting international standards to the regional context.

### 3.2 Identifying high-risk biochemical recurrence patients

#### 3.2.1 High-risk PCa: definition and choice of therapy

There is no agreement on the optimal identification of high-risk PCa patients (10–13). The panel agreed on defining high-risk PCa as PSA level > 20 ng/ml or Gleason Score (GS) >7 (International Society of Urological Pathology [ISUP] grade 4/5) or cT3a (Strong agreement, 87.5%). Most experts agreed that a PSA reading of >20 ng/mL alone is insufficient to indicate high-risk PCa (Slight agreement, 62.5%), as it may be affected by other factors (14, 15). Meanwhile, cT3a (extracapsular extension) is considered a definite indicator. For very high- or high-risk patients, the National Comprehensive Cancer Network (NCCN) guideline recommends RP in case of localized PCa with a life expectancy over 10 years or ADT combined with RT (10). Few panelists (Slight agreement, 37.5%) agreed on differentiating management criteria for PCa patients with elevated PSA levels (>20 ng/mL) as the sole risk factor from those with all risk factors. They suggested initiating RP in patients with only a PSA level >20 ng/mL; meanwhile, patients with all risk factors should undergo a multidisciplinary team (MDT) evaluation, highlighting the importance of using the MDT approach in all patients with high-risk or borderline features. The advisors recommended RP for patients with cT2b, GS 7-8, and PSA 10–20 ng/ml, in addition to very-high or high-risk patients with localized PCa and a life expectancy over 10 years (10).

#### 3.2.2 High-risk biochemical recurrence and the role of different stratification tools

##### 3.2.2.1 Prostate-specific antigen

Various factors have been discussed to identify men at high risk of BCR following RP or RT. PSA is not PCa-specific, and false positive results are a concern. Therefore, BCR is primarily detected by a serial PSA measurement to confirm the trend of rising PSA levels (13, 14). Managing BCR should involve preventing metastasis and mortality while avoiding overtreatment when possible (16). Thus, it is crucial to confirm BCR by monitoring PSA levels through repeated tests over time (Slight agreement, 62.5%).

##### 3.2.2.2 Imaging

Although conventional imaging modalities are recommended to assess BCR patients, they have limited sensitivity in detecting small-volume or early recurrent PCa (17, 18).

The role of advanced imaging modalities, such as prostate-specific membrane antigen positron emission tomography (PSMA.PET), in detecting high-risk BCR PCa was discussed because they are crucial to confirm BCR and minimize the risk of unwarranted interventions (Slight agreement, 50%). The panel emphasized the widespread adoption of Gallium (Ga) PSMA.PET/computed tomography (CT) in corroborating this state, they concurred that Ga. PSMA.PET/CT demonstrates high sensitivity and specificity for detecting recurrence (Agreement, 75%). It is also considered the most sensitive imaging modality in detecting BCR (Strong agreement, 87.5%), followed by the Fluciclovine PET/CT (Slight agreement, 37.5%). Incorporating Ga. PSMA.PET/CT in clinical practice changed the management intent of 62% of patients due to better localization of recurrence (19–21). Fluciclovine PET/CT was slightly more sensitive than other methods, with a 67.7% recurrence detection rate (22). Consequently, advanced PET/CT modalities are mostly preferred for patients with BCR who are candidates for curative salvage treatment (Strong agreement, 100%). Moreover, patients have limited access to next-generation imaging due to its unavailability in the public sector and high costs in the private domain.

##### 3.2.2.3 Doubling time and lymphadenectomies

For patients with negative Ga. PSMA.PET/CT or conventional imaging, PSA-double timing (DT) should be calculated for assessing the aggressiveness of recurrent disease and guiding treatment decisions; however, it is not routinely assessed. In the EMBARK trial, high-risk BCR patients exhibited a PSA-DT of ≤ 9 months (23). According to the EAU, high-risk BCR patients exhibited PSA-DT ≤12 months or pathologic Gleason score ≥8; while after RT, the interval to biochemical failure corresponded to ≤18 months or biopsy Gleason score ≥8 (24). Furthermore, the Egyptian Urological Guidelines state that if available, and conventional imaging is negative, consider 68Ga. PSMA.PET/CT (25). Despite the availability of user-friendly PSA-DT calculators, some advisors reported that PSA-DT is not routinely assessed in clinical practice.

**Figure 1:**
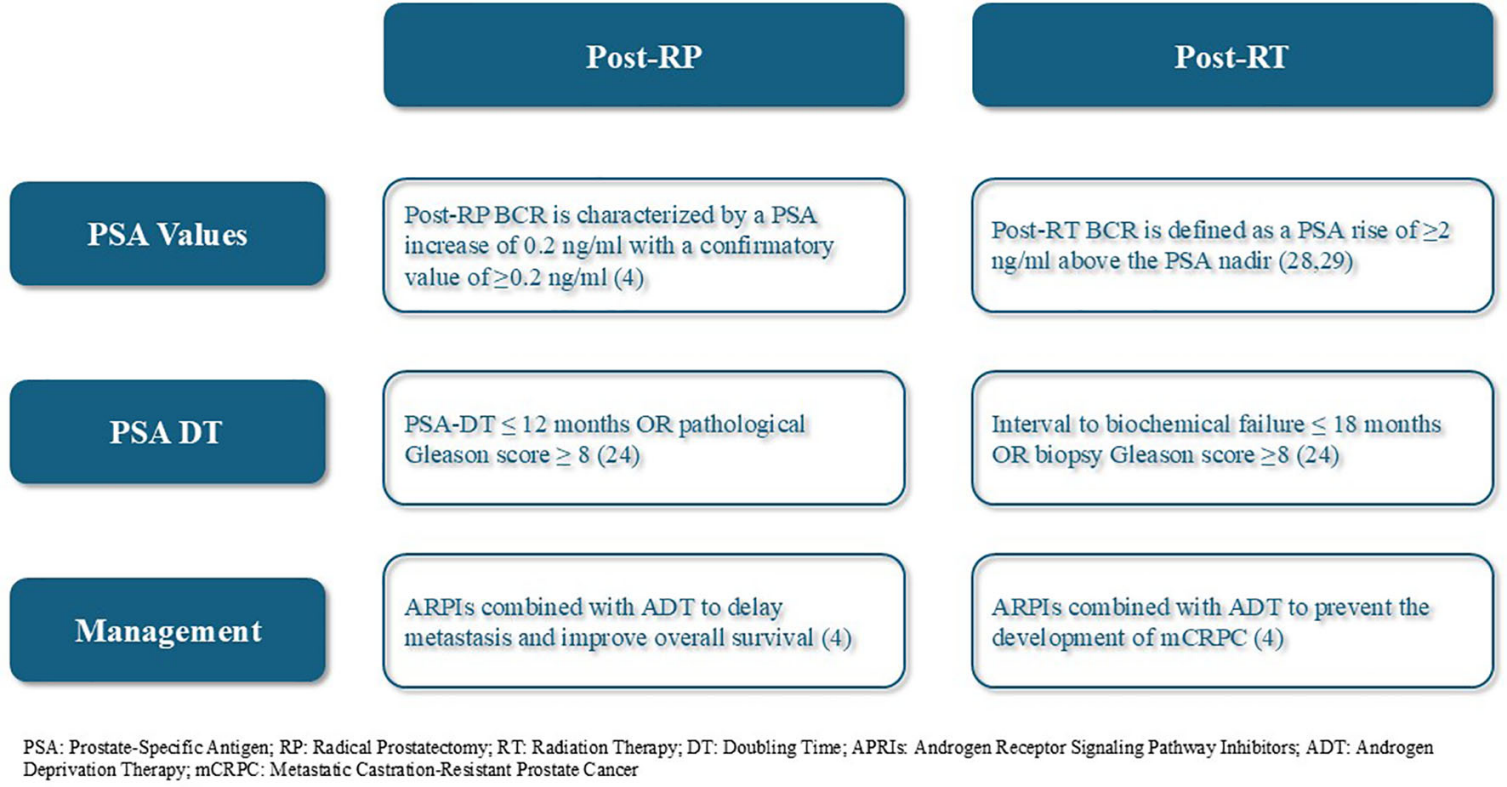
BCR Post RP and BCR Post-RT definitions and management.

When positive or negative Ga. PSMA.PET/CT, PSA-DT < 9 months, and negative conventional imaging, advisors agreed on classifying as high-risk BCR (Strong agreement, 87.5%). They advised starting short-term ADT combined with RT as salvage treatment in this case. To limit unnecessary treatment, some advisors suggested performing lymphadenectomies when Ga. PSMA.PET/CT follow-up scans were positive (26). New evidence also supports limited lymphadenectomy with negative Ga. PSMA.PET/CT (27) (**Figure 1**).

##### 3.2.2.4 Defining high-risk biochemical recurrence

Stratification of BCR into low- or high-risk is important for personalized treatment. However, the lack of clarity in existing literature leads to the advisors’ disagreement on the method of high-risk BCR identification. EAU defines BCR after RP as a PSA level ≥0.2 ng/mL with a second confirmatory PSA level ≥0.2 ng/mL (4). While NCCN uses a lower threshold of >0.1 ng/mL (5, 28). For post-RT BCR, most guidelines adopt the “Phoenix criteria” as a PSA rise of ≥2 ng/mL above the post-treatment nadir (28, 29). The advisors concurred that post-RP BCR is characterized by a PSA increase of 0.2 ng/ml with a confirmatory value of ≥0.2 ng/ml (Strong agreement, 87.5%). Similarly, post-RT BCR is defined as a PSA rise of ≥2 ng/ml above the PSA nadir (Agreement, 75%).

Advisors further agreed on the following as strong predictors of BCR classification in terms of clinical, pathological, and genomics: PSA-DT (Strong agreement, 100%), GS (Strong agreement, 87.5%), surgical margin (Agreement, 75%), clinical stage (Slight agreement, 62.5%), pre-treatment PSA value (Slight agreement, 62.5%), absolute PSA rise (Slight agreement, 50%), T stage (Slight agreement, 50%) and time to primary treatment (Slight agreement, 50%). They also agreed that genetic testing is not frequently performed in cases with strong indications, such as patients with a rapid PSA-DT and a family history of genetically driven cancers. The advisors considered patients with a PSA-DT of <3 months as metastatic, rarely achieve PSA levels <0.2 ng/mL with treatment, reflecting a poor prognosis.

The advisors agreed to describe high-risk BCR as follows: positive genomic testing, PSA-DT <1 year, GS >7, PSA > 20 ng/ml or rapidly rising, and T3/T4 staging, while PSA-DT >1 year, GS <7, T1/T2 staging, and ECOG performance (0–1) for low-risk BCR.

##### 3.2.2.5 Choice of therapy available for BCR patients

The number of patients with high-risk BCR reflects an unmet need for proper interventions after RP or RT. The incidence of BCR after primary treatment in Egypt is unknown; however, it is expected to align with global rates (27–53%) (Slight agreement, 62.5%).

The choice of therapy for managing BCR after primary PCa treatment depends on the initial therapy and the patient’s clinical characteristics. For patients experiencing BCR after RP, salvage RT (SRT), ADT, both, or active surveillance could be used (30–33). Conversely, for BCR following RT, ADT is the mainstay of treatment (34–36). Therefore, tailoring personalized treatment remains crucial to optimizing outcomes, highlighting the importance of MDT to ensure evidence-based, patient-centered care.

### 3.3 Role of androgen deprivation therapy in international guidelines, and unmet needs

#### 3.3.1 The role of ADT in managing high-risk biochemical recurrence

ADT is a cornerstone for advanced or rapidly progressing PCa, reducing PSA levels, improving progression-free survival (PFS), and extending overall survival (OS) (37–39) Nevertheless, it causes several adverse effects on several organs and systems (40–46).

Accordingly, ADT is reserved for high-risk BCR patients or those demonstrating progression (5, 47), thus it is favored in high-risk BCR with rapid PSA-DT (<6 months) (Strong agreement, 87.5%), PSA levels suggesting micrometastatic disease (detected through advanced imaging) (Agreement, 75%), and in patients originally presented with a high GS (8–10) at diagnosis (Slight Agreement, 62.5%). Conversely, the advisors disagreed on the use of ADT in the following cases: Patients at high risk of ADT side effects (Agreement, 75%), patients preferring ADT-Sparing approaches (Slight agreement, 62.5%), those with early BCR with prolonged PSA-DT (Slight agreement, 62.5%), and older patients or those with comorbidities (Slight agreement, 62.5%). Still, ADT adverse events impact the quality of life (QoL), including a threefold likely risk of depression (48).

Intermittent and continuous ADT therapy are used for advanced PCa or BCR management; however, the choice between them has been debated. Global trials have demonstrated that OS was comparable in intermittent and continuous ADT in patients with non-metastatic or BCR (38). However, intermittent ADT shows fewer adverse effects (49). Most advisors prioritized patients’ QoL, including sexual health (50, 51) and emphasized the importance of effective monotherapy for sexually active patients. They agreed to consider patients’ preferences when selecting ADT-sparing regimens (Strong agreement, 100%), while preferring continuous ADT (Slight agreement, 62.5% for continuous vs. no agreement, 25% for intermittent), likely due to its significant efficacy (49, 50, 52).

Overall, there is no agreement on optimal ADT timing for BCR or whether intermittent or continuous treatment is superior in metastatic disease. However, ADT alone is not sufficient in treating high-risk BCR PCa, leading to disease progression and mortality.

#### 3.3.2 Role of combined ADT and ARPIs in high-risk biochemical recurrence

Several trials have investigated treatment options for high-risk BCR patients (34, 53, 54). The STAMPEDE trial investigated the efficacy of combining abiraterone with enzalutamide in treating non-metastatic castration-sensitive prostate cancer (nmCSPC). However, no additional benefit compared to abiraterone monotherapy was observed (55). In 2023, the EMBARK trial evaluated the addition of enzalutamide with ADT, compared to ADT alone, in high-risk BCR patients, demonstrating significant improvements in metastasis-free survival (MFS) in the combination group (23).

The PRESTO trial evaluated the efficacy of apalutamide, a second-generation ARPI, in delaying disease progression for men with high-risk BCR. Apalutamide has shown potential improvement in PFS, offering a promising therapeutic option (56). Ongoing studies are conducted to evaluate these results further and define ARPIs’ role in clinical practice, in addition to the ongoing analyses from the EMBARK trial (57, 58). According to the European Association of Urology (EAU) guidelines, ARPIs combined with ADT are used for high-risk post-RP BCR patients to delay metastasis and improve OS, while in post-RT BCR, they are used to prevent the development of metastatic castration-resistant prostate cancer (mCRPC) (4). Advisors agreed that this combination is the first-line treatment for non-metastatic hormone-sensitive prostate cancer (nmHSPC) with high-risk BCR (Strong agreement, 87.5%). Thus, they think it’s the ideal treatment regimen after RP combines RT with short-term ADT in Egypt.

#### 3.3.3 Evaluating the role of ARPIs as monotherapy in BCR patients

NCCN-EAU-European Association of Nuclear Medicine (EANM)-European Society for Radiotherapy and Oncology (ESTRO)- European Society of Urogenital Radiology (ESUR)-ISUP-International Society of Geriatric Oncology (SIOG) guidelines recommend the use of ARPIs in this setting (4, 11, 16). The panelists emphasized that the EMBARK trial positively changed practice (Agreement, 75%) by substantiating the role of ARPIs in BCR patients. For instance, enzalutamide monotherapy provided statistically and clinically significant improvement in MFS (59, 60).

Experts highlighted several factors influencing the use of enzalutamide monotherapy in high-risk BCR, particularly focusing on patient preference and tolerability. Many patients, especially younger and fitter individuals, prefer monotherapy to avoid sexual dysfunction, hot flashes, and the side effects of ADT, aiming to preserve QoL. This treatment is also favored by those who are intolerant of ADT or concerned about testosterone depletion, as it offers a manageable alternative. Providers often prefer to avoid ADT-related adverse effects, making enzalutamide monotherapy an appealing option for improving patient adherence and maintaining overall well-being. However, advisors expressed concerns about the lack of data on OS for patients using enzalutamide monotherapy. Later in 2025, data were published and demonstrated superior OS in the combination arm (39).

In the meeting, the advisors reviewed three patient profiles for post-RP BCR patients and two other patient profiles for post-RT BCR and agreed on specific treatment plans for each:

##### 3.3.3.1 After radical prostatectomy (RP): prostate-specific antigen (PSA) >0.2 ng/ml

- Patient profile 1: PSA level < 0.5 ng/ml and PSA-DT <6 months. The first line/standard of care therapy chosen in nmHSPC patients with high-risk BCR, advisors agreed on treatment with salvage External Beam Radiation Therapy (EBRT)+ADT (Agreement, 75%),
- Patient profile 2: Genomic classifier score high-risk, GS 8–10, Life expectancy ≥10 years, PreEBRT PSA level ≥0.7 ng/ml & positive surgical margin. From a risk-adaptive treatment approach, advisors agreed on using salvage EBRT+ADT in post-RP BCR patients (Strong agreement, 87.5%)
- Patient profile 3: PSA-DT ≤12 months, GS >7, Life expectancy >10 years. The advisors recommended salvage EBRT+ADT (Slight agreement, 50%).

##### 3.3.3.2 After radiation therapy (RT): prostate-specific antigen (PSA) >2 ng/ml.

- Patient profile 1: Interval to BCR ≤18 months, GS 8–10, Life expectancy ≤10 years. The advisors recommended ARPIs + ADT (Slight agreement, 50%).

### 3.4 Examining the impact of a multidisciplinary approach

MDT is vital in diagnosing and managing PCa, including various medical fields, such as oncology, urology, radiology, and pathology. MDT helps tailor patient-specific treatment; however, it can provide conflicting opinions, prolong decision-making, and delay protocol development.

Research indicates debates about MDT involvement in early diagnosis, with urologists primarily responsible, while oncologists and radiologists play larger roles during treatment.

The experts agreed that MDT contributes to early detection (Strong agreement, 87.5%), diagnosis (Strong agreement, 100%), treatment (Strong agreement, 100% agreement), and follow-up (Strong agreement, 100%) of PCa. Experts also agreed on the urologists’ role in early detection (Strong agreement, 100%), urologists and radiologists in diagnosis, and urologists and oncologists in treatment and follow-up (Strong agreement, 100%). Thus, the advisors recommended MDT implementation for all patients, though its necessity is debated in early diagnosis. In Egypt, some entities have established MDTs, such as Ain Shams University Hospital. To implement urology MDTs nationally, MDT practice should be mandated by national guidance and public health service providers. As an example, in the national breast cancer program, to ensure MDT implementation for all patients, reimbursement for patient services was linked to MDT discussion of the case and uploading outcomes to the central official national committee. This approach could also be extended to the urology MDT.

### 3.5 Identifying associated costs and resource utilization

Metastatic PCa, linked to high costs and poor quality of life, necessitates early treatment as the disease metastasis is associated with increased healthcare resource utilization (HCRU) and overall costs (61, 62). Thus, the main treatment goals are to delay disease progression and prolong survival. Oncological diseases, including metastatic PCa, have a huge economic burden on the Egyptian healthcare system due to the increased costs of progression, follow-up, and productivity loss. This highlights the necessity of early treatment of these patients to save costs and lighten the burden of disease on the patient, society, and economy (63, 64).

According to the panel, treatment costs significantly affect treatment adherence and QoL (63). Moreover, in the healthcare sector, rising costs deplete resources and affect patient care. Thus, identifying costs is essential to spot the healthcare sector’s weak points. The panel agreed that delaying metastasis allows more effective treatment options and better patient outcomes (Strong agreement, 100%). Also, delayed metastasis is beneficial for patients’ OS.

The high-risk BCR patients’ stratification results in direct and indirect costs of medical intervention. Advisors could not reach an agreement on the high direct cost. This is due to the variation in the cost of services between different providers, and on other occasions due to the unavailability in the public domain and limitations in the private sector. Nevertheless, some services were suggested, for example, imaging, particularly PSMA PET and FDG PET, which are not reimbursed across all public sectors. Other examples include prolonged ADT and novel therapies (No agreement, 25%). An agreement was reached on the definition of moderate direct, including follow-ups, imaging, and some advanced treatments (Agreement, 75%) and indirect costs, including burden on daily life and work (Strong agreement, 100%). However, the lack of data in Egypt hindered the cost-benefit estimation. Therefore, patients’ OS should be assessed, and economic evaluations should be conducted, to help guide future budget allocation to the most effective treatment (**Table 2**).

**Table 2:** Expert panel recommendations summary.

Topic	Recommendations
Current initiatives for local guidelines and barriers	The panel suggests establishing a national cancer registry to encourage full documentation essential for addressing gaps and adapting international standards to the regional context.
BCR risk classification	When positive or negative Ga. PSMA.PET/CT, PSA-DT < 9 months, and negative conventional imaging, advisors agreed on classifying it as high-risk BCR. They advised starting short-term ADT combined with RT as salvage treatment in this case. To limit unnecessary treatment, some advisors suggested performing lymphadenectomies when Ga. PSMA.PET/CT follow-up scans were positive
	Advisors further agreed on the following as strong predictors of BCR classification in terms of clinical, pathological, and genomics: PSA-DT, GS, surgical margin, clinical stage, pre-treatment PSA value, absolute PSA rise, T stage, and time to primary treatment.
High-risk vs. low-risk BCR definitions	The advisors agreed to describe high-risk BCR as follows: positive genomic testing, PSA-DT <1 year, GS >7, PSA > 20 ng/ml or rapidly rising, and T3/T4 staging, while PSA-DT >1 year, GS <7, T1/T2 staging, and ECOG performance (0–1) for low-risk BCR. This agrees with some guidelines, such as EAU
ADT usage disagreements	The advisors disagreed on the use of ADT in the following cases: Patients at high risk of ADT side effects, patients preferring ADT-Sparing approaches, those with early BCR with prolonged PSA-DT, and older patients or those with comorbidities.
Patient QoL and ADT preferences	Most advisors prioritized patients’ QoL, including sexual health, and emphasized the importance of effective monotherapy for sexually active patients. They agreed to consider patients’ preferences when selecting ADT-sparing regimens, while preferring continuous ADT.
ADT limitations in high-risk BCR	ADT alone is not sufficient in treating high-risk BCR PCa, leading to disease progression and mortality.
Personalized treatment and MDT role	Tailoring personalized treatment remains crucial to optimizing outcomes, highlighting the importance of MDT to ensure evidence-based, patient-centered care.
MDT implementation	The advisors recommended MDT implementation for all patients, though its necessity is debated in early diagnosis.

BCR, Biochemical recurrence; ADT, Androgen Deprivation Therapy; QoL, Quality of Life; MDT, Multidisciplinary Team; ECOG, Eastern Cooperative Oncology Group; EAU, European Association of Urology; PSMA.PET/CT, Prostate-Specific Membrane Antigen Positron Emission Tomography/Computed Tomography.

## 4 Unmet questions and future directions

The effectiveness of treatment, the impact of early intervention, the role of early systemic therapy, and the potential benefits compared to risks of treatment remain unresolved issues, with key questions including whether aggressive or selective treatment is more beneficial or risk-effective.

## 5 Conclusion

The management of PCa is complex, requiring robust evidence to guide clinical decisions and optimal treatment timing. The balance between potential adverse events and benefits is crucial, with further studies needed to evaluate this relationship in depth. This narrative, evidence-supported expert-opinion review emphasizes the necessity of personalized treatment methods and encourages further research to enhance diagnosis and management strategies.

## References

[1] Rawla P . Epidemiology of prostate cancer. World J Oncol. (2019) 10:63–89. doi: 10.14740/wjon1191, PMID: 31068988 PMC6497009

[2] Cancer today. Available online at: https://gco.iarc.who.int/today/en (Accessed November 27, 2025).

[3] Simon NI Parker C Hope TA Paller CJ . Best approaches and updates for prostate cancer biochemical recurrence. Am Soc Clin Oncol Educ Book. (2022) 42):352–9. doi: 10.1200/EDBK_351033, PMID: 35503984 PMC9844546

[4] Van Den Broeck T Van Den Bergh RCN Briers E Cornford P Cumberbatch M Tilki D . Biochemical recurrence in prostate cancer: the european association of urology prostate cancer guidelines panel recommendations. Eur Urol Focus. (2020) 6:231–4. doi: 10.1016/j.euf.2019.06.004, PMID: 31248850

[5] Xu H Zhu Y Dai B Ye DW . National Comprehensive Cancer Network (NCCN) risk classification in predicting biochemical recurrence after radical prostatectomy: A retrospective cohort study in Chinese prostate cancer patients. Asian J Androl. (2018) 20:551–4. doi: 10.4103/aja.aja_52_18, PMID: 30027928 PMC6219292

[6] Roach M Hanks G Thames H Schellhammer P Shipley WU Sokol GH . Defining biochemical failure following radiotherapy with or without hormonal therapy in men with clinically localized prostate cancer: Recommendations of the RTOG-ASTRO Phoenix Consensus Conference. Int J Radiat Oncol Biol Phys. (2006) 65:965–74. doi: 10.1016/j.ijrobp.2006.04.029, PMID: 16798415

[7] Malone S Shayegan B Basappa NS Chi K Conter HJ Hamilton RJ . Management algorithms for metastatic prostate cancer. Can Urological Assoc J. (2019) 13:50. doi: 10.5489/cuaj.5840, PMID: 31039111 PMC7012295

[8] EAU guidelines on prostate cancer. Available online at: https://uroweb.org/guidelines/prostate-cancer (Accessed November 27, 2025).

[9] EUG. Available online at: http://eug-eg.net/guidelinesmethodology (Accessed November 27, 2025).

[10] Parker C Castro E Fizazi K Heidenreich A Ost P Procopio G . Prostate cancer: ESMO Clinical Practice Guidelines for diagnosis, treatment and follow-up. Ann Oncol. (2020) 31:1119–34. doi: 10.1016/j.annonc.2020.06.011, PMID: 32593798

[11] Schaeffer EM Srinivas S Adra N An Y Barocas D Bitting R . Prostate cancer, version 4.2023, NCCN clinical practice guidelines in oncology. J Natl Compr Cancer Network. (2023) 21:1067–96. doi: 10.6004/jnccn.2023.0050, PMID: 37856213

[12] Eastham JA Auffenberg GB Barocas DA Chou R Crispino T Davis JW . Clinically localized prostate cancer: AUA/ASTRO guideline, part I: introduction, risk assessment, staging, and risk-based management. J Urol. (2022) 208:10–8. doi: 10.1097/JU.0000000000002757, PMID: 35536144

[13] Bekelman JE Rumble RB Chen RC Pisansky TM Finelli A Feifer A . Clinically localized prostate cancer: ASCO clinical practice guideline endorsement of an american urological association/american society for radiation oncology/society of urologic oncology guideline. J Clin Oncol. (2018) 36:3251–8. doi: 10.1200/JCO.18.00606, PMID: 30183466

[14] Lumbreras B Parker LA Caballero-Romeu JP Gómez-Pérez L Puig-García M López-Garrigós M . Variables associated with false-positive PSA results: A cohort study with real-world data. Cancers. (2022) 15:261. doi: 10.3390/cancers15010261, PMID: 36612257 PMC9818944

[15] Aigbe E Irekpita E Ogbetere F Alili U . Correlation between prostate volume and prostate-specific antigen in Nigerian men with symptomatic histologically-diagnosed benign prostatic hyperplasia. Niger J Clin Pract. (2022) 25:1523–8. doi: 10.4103/njcp.njcp_67_22, PMID: 36149214

[16] Cornford P van den Bergh RCN Briers E Van den Broeck T Brunckhorst O Darraugh J . EAU-EANM-ESTRO-ESUR-ISUP-SIOG guidelines on prostate cancer—2024 update. Part I: screening, diagnosis, and local treatment with curative intent. Eur Urol. (2024) 86:148–63. doi: 10.1016/j.eururo.2024.03.027, PMID: 38614820

[17] Parent EE Schuster DM . Update on 18F-fluciclovine PET for prostate cancer imaging. J Nucl Med. (2018) 59:733–9. doi: 10.2967/jnumed.117.204032, PMID: 29523631 PMC6910635

[18] Overview | Prostate cancer: diagnosis and management. NICE. (2021). (Accessed November 27, 2025).

[19] Bach-Gansmo T Nanni C Nieh PT Zanoni L Bogsrud TV Sletten H . Multisite experience of the safety, detection rate and diagnostic performance of fluciclovine (^18^ F) positron emission tomography/computerized tomography imaging in the staging of biochemically recurrent prostate cancer. J Urol. (2017) 197:676–83. doi: 10.1016/j.juro.2016.09.117, PMID: 27746282 PMC5645081

[20] Morigi JJ Stricker PD Van Leeuwen PJ Tang R Ho B Nguyen Q . Prospective comparison of^18^ F-fluoromethylcholine versus^68^ ga-PSMA PET/CT in prostate cancer patients who have rising PSA after curative treatment and are being considered for targeted therapy. J Nucl Med. (2015) 56:1185–90. doi: 10.2967/jnumed.115.160382, PMID: 26112024

[21] Roach PJ Francis R Emmett L Hsiao E Kneebone A Hruby G . The impact of 68Ga-PSMA PET/CT on management intent in prostate cancer: results of an Australian prospective multicenter study. J Nucl Med. (2018) 59:82–8. doi: 10.2967/jnumed.117.197160, PMID: 28646014

[22] Afshar-Oromieh A Zechmann CM Malcher A Eder M Eisenhut M Linhart HG . Comparison of PET imaging with a 68Ga-labelled PSMA ligand and 18F-choline-based PET/CT for the diagnosis of recurrent prostate cancer. Eur J Nucl Med Mol Imaging. (2014) 41:11–20. doi: 10.1007/s00259-013-2525-5, PMID: 24072344 PMC3843747

[23] Shore ND . LBA02–09 EMBARK: A phase 3 randomized study of enzalutamide or placebo plus leuprolide acetate and enzalutamide monotherapy in high-risk biochemically recurrent prostate cancer. J Urol. (2023) 210:224–6. doi: 10.1097/JU.0000000000003518, PMID: 37119051

[24] Santiago I Gómez Rivas J . Re: EAU-EANM-ESTRO-ESUR-ISUP-SIOG Guidelines on Prostate Cancer. Eur Urol. (2026) 89:100–2. doi: 10.1016/j.eururo.2025.07.023, PMID: 40813208

[25] Rashed A Rageb M Hussein A Elmekaty K Elbashir S Zahran M . Egypian Urological Guidelines Book, 2021. Committee XIV: Male Infertility. The Egyptian Medicine (2021). Available online at: https://www.ema1920.org.eg/ (Accessed November 27, 2025).

[26] EAU 2024: negative staging PSMA in high-risk disease: can we skip extended pelvic lymph node dissection? No, we cannot. Available online at: https://www.urotoday.com/conference-highlights/eau-2024/eau-2024-prostate-cancer/151088-eau-2024-negative-staging-psma-in-high-risk-disease-can-we-skip-extended-plnd-no-we-cannot.html/ (Accessed November 27, 2025).

[27] Dong B Zhan H Luan T Wang J . The role and controversy of pelvic lymph node dissection in prostate cancer treatment: a focused review. World J Surg Oncol. (2024) 22:68–. doi: 10.1186/s12957-024-03344-2, PMID: 38403658 PMC10895790

[28] Lowrance W Dreicer R Jarrard DF Scarpato KR Kim SK Kirkby E . Updates to advanced prostate cancer: AUA/SUO guideline (2023). J Urol. (2023) 209:1082–90. doi: 10.1097/JU.0000000000003452, PMID: 37096583

[29] Jansen BHE Van Leeuwen P Wondergem M Van Der Sluis T Nieuwenhuijzen J Knol R . The Phoenix criteria for biochemically recurrent prostate cancer after curative radiotherapy appear obsolete in the era of prostate-specific membrane antigen PET: A plea for urgent re-evaluation of current guidelines. Eur Urol Suppl. (2019) 18:e3408–e3409. Available online at: https://uroonco.uroweb.org/publication/the-phoenix-criteria-for-biochemicallyrecurrent-prostate-cancer-after-curative-radiotherapy-appear-obsolete-in-the-era-ofprostate-specific-membrane-antigen-pet-a-plea-for-urgent-re-evaluation-of-cu/ (Accessed November 27, 2025).

[30] Pazona JF Han M Hawkins SA Roehl KA Catalona WJ . SALVAGE RADIATION THERAPY FOR PROSTATE SPECIFIC ANTIGEN PROGRESSION FOLLOWING RADICAL PROSTATECTOMY: 10-YEAR OUTCOME ESTIMATES. J Urol. (2005) 174:1282–6. doi: 10.1097/01.ju.0000173911.82467.f9, PMID: 16145393

[31] Pisansky TM Thompson IM Valicenti RK D’Amico AV Selvarajah S . Adjuvant and salvage radiotherapy after prostatectomy: Astro/aua guideline amendment 2018-2019. J Urol. (2019) 202:533–8. doi: 10.1097/JU.0000000000000295, PMID: 31042111 PMC8680266

[32] Freedland SJ De Almeida Luz M De Giorgi U Gleave M Gotto GT Pieczonka CM . Improved outcomes with enzalutamide in biochemically recurrent prostate cancer. New Engl J Med. (2023) 389:1453–65. doi: 10.1056/NEJMoa2303974, PMID: 37851874

[33] Garcia-Albeniz X Chan JM Paciorek A Logan RW Kenfield SA Cooperberg MR . Immediate versus deferred initiation of androgen deprivation therapy in prostate cancer patients with PSA-only relapse. An observational follow-up study. Eur J Cancer. (2015) 51:817–24. doi: 10.1016/j.ejca.2015.03.003, PMID: 25794605 PMC4402138

[34] Valle LF Lehrer EJ Markovic D Elashoff D Levin-Epstein R Karnes RJ . A systematic review and meta-analysis of local salvage therapies after radiotherapy for prostate cancer (MASTER). Eur Urol. (2021) 80:280–92. doi: 10.1016/j.eururo.2020.11.010, PMID: 33309278 PMC10262981

[35] Lei JH Liu LR Wei Q Yan SB Song TR Lin FS . Systematic review and meta-analysis of the survival outcomes of first-line treatment options in high-risk prostate cancer. Sci Rep. (2015) 5:7713. doi: 10.1038/srep07713, PMID: 25578739 PMC5378991

[36] Matsukawa A Yanagisawa T Fazekas T Miszczyk M Tsuboi I Kardoust Parizi M . Salvage therapies for biochemical recurrence after definitive local treatment: a systematic review, meta-analysis, and network meta-analysis. Prostate Cancer Prostatic Dis. (2024) 28:610–22. doi: 10.1038/s41391-024-00890-4, PMID: 39266730 PMC12399422

[37] Duchesne GM Woo HH Bassett JK Bowe SJ D’Este C Frydenberg M . Timing of androgen-deprivation therapy in patients with prostate cancer with a rising PSA (TROG 03.06 and VCOG PR 01–03 [TOAD]): a randomised, multicentre, non-blinded, phase 3 trial. Lancet Oncol. (2016) 17:727–37. doi: 10.1016/S1470-2045(16)00107-8, PMID: 27155740

[38] Crook JM O’Callaghan CJ Duncan G Dearnaley DP Higano CS Horwitz EM . Intermittent androgen suppression for rising PSA level after radiotherapy. New Engl J Med. (2012) 367:895–903. doi: 10.1056/NEJMoa1201546, PMID: 22931259 PMC3521033

[39] Shore ND Luz M de A De Giorgi U Gleave M Gotto GT Pieczonka CM . Improved survival with enzalutamide in biochemically recurrent prostate cancer. New Engl J Med. (2025). doi: 10.1056/NEJMoa2510310, PMID: 41124201

[40] Nguyen PL Chen MH Beckman JA Beard CJ Martin NE Choueiri TK . Influence of androgen deprivation therapy on all-cause mortality in men with high-risk prostate cancer and a history of congestive heart failure or myocardial infarction. Int J Radiat OncologyBiologyPhysics. (2012) 82:1411–6. doi: 10.1016/j.ijrobp.2011.04.067, PMID: 21708431

[41] Lee HY Chen HL Teoh JYC Chen TC Hao SY Tsai HY . Abiraterone and enzalutamide had different adverse effects on the cardiovascular system: a systematic review with pairwise and network meta-analyses. Prostate Cancer Prostatic Dis. (2020) 24:244–52. doi: 10.1038/s41391-020-00275-3, PMID: 32860011

[42] Spry NA Galvão DA Davies R La Bianca S Joseph D Davidson A . Long-term effects of intermittent androgen suppression on testosterone recovery and bone mineral density: Results of a 33-month observational study. BJU Int. (2009) 104:806–12. doi: 10.1111/j.1464-410X.2009.08458.x, PMID: 19281463

[43] Harrington JM Schwenke DC Epstein DR Bailey DE . Androgen-deprivation therapy and metabolic syndrome in men with prostate cancer. Oncol Nurs Forum. (2014) 41:21–9. doi: 10.1188/14.ONF.21-29, PMID: 24368236

[44] Donovan KA Walker LM Wassersug RJ Thompson LMA Robinson JW . Psychological effects of androgen-deprivation therapy on men with prostate cancer and their partners. Cancer. (2015) 121:4286–99. doi: 10.1002/cncr.29672, PMID: 26372364

[45] Mohile SG Mustian K Bylow K Hall W Dale W . Management of complications of androgen deprivation therapy in the older man. Crit Rev Oncol Hematol. (2009) 70:235–55. doi: 10.1016/j.critrevonc.2008.09.004, PMID: 18952456 PMC3074615

[46] Donovan KA Gonzalez BD Nelson AM Fishman MN Zachariah B Jacobsen PB . Effect of androgen deprivation therapy on sexual function and bother in men with prostate cancer: A controlled comparison. Psychooncology. (2018) 27:316–24. doi: 10.1002/pon.4463, PMID: 28557112 PMC5709275

[47] Van Den Bergh RCN Van Casteren NJ Van Den Broeck T Fordyce ER Gietzmann WKM Stewart F . Role of hormonal treatment in prostate cancer patients with nonmetastatic disease recurrence after local curative treatment: A systematic review. Eur Urol. (2016) 69:802–20. doi: 10.1016/j.eururo.2015.11.023, PMID: 26691493

[48] Thomas HR Chen MH D’Amico AV Bennett CL Kattan MW Sartor O . Association between androgen deprivation therapy and patient-reported depression in men with recurrent prostate cancer. Clin Genitourin Cancer. (2018) 16:313–7. doi: 10.1016/j.clgc.2018.05.007, PMID: 29866496

[49] Da Silva FC Da Silva FMC Gonçalves F Santos A Kliment J Whelan P . Locally advanced and metastatic prostate cancer treated with intermittent androgen monotherapy or maximal androgen blockade: results from a randomised phase 3 study by the south european uroncological group. Eur Urol. (2014) 66:232–9. doi: 10.1016/j.eururo.2013.03.055, PMID: 23582949

[50] Gay HA Sanda MG Liu J Wu N Hamstra D Wei JT . External beam radiation therapy or brachytherapy with or without short-course neoadjuvant androgen deprivation therapy: results of a multicenter, prospective study of quality of life. Int J Radiat Oncol Biol Phys. (2017) 98:304–17. doi: 10.1016/j.ijrobp.2017.02.019, PMID: 28463150 PMC5493021

[51] Fode M Sønksen J . Sexual function in elderly men receiving androgen deprivation therapy (ADT). Sex Med Rev. (2014) 2:36–46. doi: 10.1002/smrj.17, PMID: 27784542

[52] Tsai HT Penson DF Makambi KH Lynch JH Van Den Eeden SK Potosky AL . Efficacy of intermittent androgen deprivation therapy vs conventional continuous androgen deprivation therapy for advanced prostate cancer: A meta-analysis. Urology. (2013) 82:327–34. doi: 10.1016/j.urology.2013.01.078, PMID: 23896094 PMC4154705

[53] Attard G Murphy L Clarke NW Cross W Jones RJ Parker CC . Abiraterone acetate and prednisolone with or without enzalutamide for high-risk non-metastatic prostate cancer: a meta-analysis of primary results from two randomised controlled phase 3 trials of the STAMPEDE platform protocol. Lancet. (2022) 399:447–60. doi: 10.1016/S0140-6736(21)02437-5, PMID: 34953525 PMC8811484

[54] Morgans AK Gschwend JE Shore ND Ross A Feng F Hope T . 1841TiP Darolutamide plus androgen-deprivation therapy (ADT) in patients with high-risk biochemical recurrence (BCR) of prostate cancer: A phase III, randomized, double-blind, placebo-controlled study (ARASTEP). Ann Oncol. (2023) 34:S997–8. doi: 10.1016/j.annonc.2023.09.2789

[55] Parker CC James ND Brawley CD Clarke NW Hoyle AP Ali A . Radiotherapy to the primary tumour for newly diagnosed, metastatic prostate cancer (STAMPEDE): a randomised controlled phase 3 trial. Lancet. (2018) 392:2353–66. doi: 10.1016/S0140-6736(18)32486-3, PMID: 30355464 PMC6269599

[56] Aggarwal R Heller G Hillman DW Xiao H Picus J Taplin ME . PRESTO: A phase III, open-label study of intensification of androgen blockade in patients with high-risk biochemically relapsed castration-sensitive prostate cancer (AFT-19). J Clin Oncol. (2024) 42:1114–23. doi: 10.1200/JCO.23.01157, PMID: 38261983 PMC11637124

[57] ASCO GU 2024: ARASTEP: darolutamide + ADT in patients with high-risk BCR of prostate cancer: A phase 3, randomized, double-blind, placebo-controlled study. Available online at: https://www.urotoday.com/conference-highlights/asco-gu-2024/asco-gu-2024-prostate-cancer/149349-asco-gu-2024-arastep-darolutamide-adt-in-patients-with-high-risk-bcr-of-prostate-cancer-a-phase-3-randomized-double-blind-placebo-controlled-study.html (Accessed November 27, 2025).

[58] Treatment intensification for high-risk biochemically recurrent M0 HSPC: EMBARK in the PSMA PET era and ongoing trials. Available online at: https://www.urotoday.com/library-resources/mhspc/156960-treatment-intensification-for-high-risk-biochemically-recurrent-m0-hspc-embark-in-the-psma-pet-era-and-ongoing-trials.html (Accessed November 27, 2025).

[59] Laccetti AL Smith MR Scher HI Verholen F Adorjan P Dissanayake M . ARAMON: A phase 2, randomized, open-label study comparing darolutamide (DARO) vs enzalutamide (ENZA) monotherapy on serum testosterone levels in patients (pts) with castration-sensitive prostate cancer (CSPC) after biochemical recurrence (BCR). J Clin Oncol. (2024) 42:TPS243–3. doi: 10.1200/JCO.2024.42.4_suppl.TPS243

[60] EAU 2024: negative staging PSMA in high-risk disease: can we skip extended pelvic lymph node dissection? No, we cannot. Available online at: https://www.urotoday.com/conference-highlights/eau-2024/eau-2024-prostate-cancer/151088-eau-2024-negative-staging-psma-in-high-risk-disease-can-we-skip-extended-plnd-no-we-cannot.html (Accessed November 27, 2025).

[61] Ko GC Hansen R Carlson J . Comparing costs and health care resource utilization between nmHSPC and mHSPC patients: a retrospective claims analysis. J Manag Care Spec Pharm. (2022) 28:287–95. doi: 10.18553/jmcp2022283287, PMID: 35199577 PMC10372963

[62] Svensson J Lissbrant IF Gauffin O Hjälm-Eriksson M Kilany S Fagerlund K . Time spent in hormone-sensitive and castration-resistant disease states in men with advanced prostate cancer, and its health economic impact: registry-based study in Sweden. Scand J Urol. (2021) 55:1–8. doi: 10.1080/21681805.2020.1851762, PMID: 33300403

[63] Soliman R Oke J Sidhom I Bhakta N Bolous NS Tarek N . Cost-effectiveness of childhood cancer treatment in Egypt: Lessons to promote high-value care in a resource-limited setting based on real-world evidence. EClinicalMedicine. (2023) 55:101729. doi: 10.1016/j.eclinm.2022.101729, PMID: 36386036 PMC9646894

[64] Elsisi GH El-Attar MM Ismaeil SM El-Shater MES Kirollos MG Sedrak AS . Estimation of prostate cancer cost in Egypt from a societal perspective. Global J Qual Saf Healthcare. (2023) 6:33–41. doi: 10.36401/JQSH-22-20, PMID: 37333760 PMC10275631

